# Variation in duration of repeat prescriptions: a primary care cohort study in England

**DOI:** 10.3399/BJGP.2024.0326

**Published:** 2025-06-27

**Authors:** Brian MacKenna, Andrew D Brown, Richard Croker, Alex J Walker, Ben Goldacre, Apostolos Tsiachristas, Dave Evans, Peter Inglesby, Seb Bacon, Helen J Curtis

**Affiliations:** Nuffield Department of Primary Care Health Sciences (NDPCHS), University of Oxford, Oxford, UK; Bennett Institute for Applied Data Science, NDPCHS, University of Oxford, Oxford, UK; Nuffield Department of Primary Care Health Sciences (NDPCHS), University of Oxford, Oxford, UK; Bennett Institute for Applied Data Science, NDPCHS, University of Oxford, Oxford, UK; Nuffield Department of Primary Care Health Sciences (NDPCHS), University of Oxford, Oxford, UK; Bennett Institute for Applied Data Science, NDPCHS, University of Oxford, Oxford, UK; Nuffield Department of Primary Care Health Sciences (NDPCHS), University of Oxford, Oxford, UK; Bennett Institute for Applied Data Science, NDPCHS, University of Oxford, Oxford, UK; Nuffield Department of Primary Care Health Sciences (NDPCHS), University of Oxford, Oxford, UK; Bennett Institute for Applied Data Science, NDPCHS, University of Oxford, Oxford, UK; Nuffield Department of Primary Care Health Sciences (NDPCHS), University of Oxford, Oxford, UK; Department of Psychiatry, University of Oxford, Oxford, UK; Nuffield Department of Primary Care Health Sciences (NDPCHS), University of Oxford, Oxford, UK; Bennett Institute for Applied Data Science, NDPCHS, University of Oxford, Oxford, UK; Nuffield Department of Primary Care Health Sciences (NDPCHS), University of Oxford, Oxford, UK; Bennett Institute for Applied Data Science, NDPCHS, University of Oxford, Oxford, UK; Nuffield Department of Primary Care Health Sciences (NDPCHS), University of Oxford, Oxford, UK; Bennett Institute for Applied Data Science, NDPCHS, University of Oxford, Oxford, UK; Nuffield Department of Primary Care Health Sciences (NDPCHS), University of Oxford, Oxford, UK; Bennett Institute for Applied Data Science, NDPCHS, University of Oxford, Oxford, UK

**Keywords:** chronic disease, electronic health records, primary care, repeat prescription, routine medication, statins

## Abstract

**Background:**

Many patients receive repeat prescriptions for routine medications used to treat chronic conditions. Doctors typically issue repeat prescriptions with durations ranging from 28 to 84 days. There is currently no national guidance in England for the optimal prescription duration for routine medications.

**Aim:**

To evaluate current prescription durations for five common routine medications in England; explore and visualise geographical variation; and identify practice factors that are associated with shorter prescribing duration to inform policy making.

**Design and setting:**

A retrospective cohort study of NHS primary care prescribing data in England from December 2018 to November 2019.

**Method:**

The prescription duration was analysed for five common routine medications in England; ramipril, atorvastatin, simvastatin, levothyroxine, and amlodipine. Variation was assessed between regional clinical commissioning groups (CCGs), and practice factors associated with different durations were identified.

**Results:**

Of the common medications included, 28-day prescriptions accounted for 48.5% (2.5 billion) tablets/capsules issued, while 43.6% were issued for 56 days. There was very wide regional variation (7.2%–95.0%) in the proportion of 28-day prescriptions issued by CCGs. Practice dispensing status was the most likely predictor of prescription duration; dispensing practices had a higher 28-day prescribing proportion than non-dispensing practices. The proportion of patients with chronic conditions and the electronic health record system used by a practice were also associated with prescription duration.

**Conclusion:**

This analysis of OpenPrescribing data showed that repeat prescriptions of 28 days are common for patients taking routine medications for chronic conditions, particularly in dispensing practices. This provides data to inform the policy debate on current practice. Configuration of electronic health record systems offer an opportunity to implement and evaluate new policies on repeat prescription duration in England.

## Introduction

General practices in England issue 1.1 billion prescriptions every year,^1^ two-thirds of which are estimated to be repeat prescriptions for routine medications commonly used to treat chronic conditions.^2^ However, NHS England (see Table 1 for descriptions of healthcare organisations referred to in this article) does not issue national guidance on the duration of prescriptions, and doctors are advised to select a ‘clinically appropriate’ duration.^3^ Some clinical commissioning groups (CCGs) previously published local guidance recommending GPs to issue 28-day prescriptions to minimise wastage from unused medication.^4^ A greater proportion of longer prescriptions are issued in Scotland and Northern Ireland compared with England,^4^ and since 2022, NHS Wales has recommended repeat prescription intervals of 56 days where appropriate.^5^ Following a systematic review and economic modelling, it has previously been suggested that NHS England should recommend 3-month repeat prescriptions.^6^ This is expected to lead to improved medication adherence, reduced inconvenience for patients, and net saved costs in terms of staff time and pharmacy dispensing fees that are likely to outweigh any potential costs of unused medications.^7^–^10^

**Table 1:** NHS organisations in England discussed in this article

**Clinical commissioning groups** Clinically-led administrative NHS bodies responsible for the planning and commissioning of healthcare services for their local area, including managing the NHS budget for all prescriptions written by GPs in their membership. There were approximately 200 CCGs in England, of which all local GP practices were members. In 2022, CCGs were removed following an NHS reorganisation and their functions were taken on by 42 integrated care boards (ICBs).
**NHS Business Services Authority** An NHS body that calculates the remuneration and reimbursement due to dispensing contractors across England and publishes data on prescribing.
**NHS Digital** The national information and technology partner to the health and care system. It publishes various statistics, such as the number of patients registered at a general practice, and was responsible for procuring electronic health record systems on behalf of the NHS. From February 2023, NHS Digital was subsumed into NHS England.
**NHS England** The executive body overseeing NHS operations in England. It sets health priorities, allocates resources, and supports healthcare providers in delivering quality services. NHS England was previously responsible for commissioning primary care and specialised services, but this is now being transferred to ICBs.
**Public Health England** An executive agency responsible for protecting and improving public health in the UK until October 2021. Its functions were transferred to the UK Health Security Agency and the Office for Health Improvement and Disparities.
**Dispensing contractors** Dispensing contractors are private businesses, including community pharmacies, dispensing doctors, and appliance contractors, who are commissioned by the NHS to dispense prescriptions for medicines, appliances, and other healthcare products written in general practices. They typically receive payment to cover the cost of the item ordered and a fee for the work.
**Community pharmacies** Pharmacies that dispense prescriptions, offer health advice, and sell over-the-counter medicines to a local community. They dispense the majority of prescriptions written in primary care. They can also provide many additional services like vaccinations and health screenings, with costs reimbursed by the NHS.
**Dispensing doctors** GP practices that both prescribe and dispense medications to patients, primarily in rural areas where access to a pharmacy may be limited.

OpenPrescribing.net is an openly accessible explorer for all prescriptions dispensed in primary care in England. It was launched in 2015, and has around 20 000 unique users every month, including doctors, pharmacists, and patients. OpenPrescribing enables searches of NHS reimbursement data published by NHS Business Services Authority that has been collated from NHS dispensers. Most NHS dispensers are community pharmacies, which are separate from general practices; however, in some (mainly rural) areas, where dispensing volumes are too low to sustain a separate business, some practices have a contract to dispense their own prescriptions. OpenPrescribing displays numerous predefined standard measures for safety, cost, and effectiveness for every practice in England. This includes a measure for the proportion prescribed as 7-day durations for a subset of products, but it does not yet describe longer durations.^11^

**How this fits in**

In England, there is currently no national guidance on the optimal duration of repeat prescriptions, which account for approximately two-thirds of the 1.1 billion prescriptions issued every year. Researchers have called for a nationally recommended duration of 3 months (in practice, an 84-day prescription) to increase adherence with medication, reduce inconvenience for patients, and reduce GP and pharmacist workloads. This study presents data from openly available prescribing data to help inform policy making. The study shows that from December 2018 to November 2019, 28-day prescriptions accounted for 48.5% (2.5 billion) of tablets/capsules issued, while 43.6% were issued for 56 days, with very wide regional variation in the proportion of 28-day prescriptions (7.2%–95.0%). Practice dispensing status was the most likely predictor of prescription duration; however, the electronic health record software used by a practice was also associated with prescription duration.

The aim of this study was to analyse current prescription durations for five routine medications commonly prescribed in England; explore and visualise geographical variation; and identify practice factors associated with shorter prescribing duration to inform policy making.

## Method

### Study design

This was a retrospective observational cohort study using prescribing data from all NHS GP practices in England over 1 year, from December 2018 to November 2019.

### Data sources

Prescribing data were extracted from the OpenPrescribing.net database. This database allows searching of publicly accessible data, published by the NHS Business Services Authority, on the prescriptions dispensed each month by every prescribing organisation in NHS primary care in England since mid-2010.^12^ These monthly datasets contain one row for each strength and formulation of a medication (for example, atorvastatin 20 mg tablets) per prescribing organisation. Columns describe ‘items’ (the number of times each medication was issued on prescription), ‘total quantity’ (total unit doses issued across all items, for example, number of tablets), and ‘cost’. Crucially for this analysis, these data are split according to the ‘quantity per item’; that is, the amount of medication dispensed per prescription (for example, 28 tablets or 56 tablets). These data are sourced from community pharmacy claims and therefore contain all items that were dispensed.

Data on which electronic health record (EHR) software is used by each general practice were extracted from a monthly file, circulated by NHS Digital to interested parties and available on request^13^ (now published annually).^14^ Data on GP practice characteristics were obtained from Public Health England (now the Office for Health Improvement and Disparities).^15^

### Basket of medicines

All available prescribing data from mid-2010 to November 2019 were extracted. These were the most current data available at the time of analysis prior to the COVID-19 pandemic. A basket of medicines was identified to include commonly prescribed routine medications suitable for analysis, using a method previously described for 7-day prescribing measures on OpenPrescribing.^11^ The top 50 most commonly prescribed tablets/capsules by chemical substance were identified. The data do not include dosage instructions (for example, ‘take two tablets twice a day’), so prescribing duration cannot be accurately calculated for medications with mixed dosing regimens. Therefore, the basket of medicines was limited to those that, in the clinical experience of two senior pharmacists, matched the following criteria:

- nearly exclusively prescribed ‘once daily’ in tablet or capsule form based on *British National Formulary* dosing;
- has consistently high levels of prescribing across England; and
- are included in national recommendations for treatment or prophylaxis of common conditions.

This resulted in a basket of five medicines: ramipril, atorvastatin, simvastatin, levothyroxine, and amlodipine (see Table 2). This is a pragmatic surrogate reflecting what is possible with current technology for prescribing dose syntax and associated data. It was noted that even with medicines prescribed once daily, occasionally some patients might have an alternative dosing schedule (for example, ‘simvastatin 10 mg, take two at night’).

**Table 2:** Medications included in analysis (only tablet/capsule forms are included)

Medication	BNF code for chemical substance	Typical chronic conditions
Ramipril	0205051R0	Hypertension, cardiovascular disease prevention, renal disease, and heart failure
Atorvastatin	0212000B0	Hypercholesterolaemia and cardiovascular disease prevention
Simvastatin	0212000Y0	Hypercholesterolaemia and cardiovascular disease prevention
Levothyroxine	0602010V0	Hypothyroidism
Amlodipine	0206020A0	Hypertension and angina

BNF = British National Formulary.

### Data processing

All available data covering December 2018 to November 2019 were extracted (the most recent available 12-month period at the time of analysis) for the basket of medicines, limited to those in tablet, capsule, caplet, or equivalent forms by those containing the string ‘Tab’ or ‘Cap’ in the medication name. Only standard general practices were included in the dataset; other organisations such as walk-in centres, prisons, and hospitals (according to the NHS Digital dataset of practice characteristics) were excluded. Practices that had not prescribed any of the five selected medications were also excluded. Practices were grouped into their parent CCGs for regional analyses.

### Prescribing durations

The items issued per duration were summed and plotted on a histogram. For further analyses the data were filtered to the most common ‘quantity per item’ values, which were multiples of 28 days: 28 days (approximately 1 month), 56 days (approximately 2 months), or 84 days (approximately 3 months). Seven-day prescribing was excluded as this is more likely to be done with clinical justification or provision of ‘dosette box’,^11^ and therefore not easily amenable to switching to longer durations. The proportion of medicines issued for each duration was calculated using the total quantity (number of tablets/capsules), as the number of items cannot be compared directly (that is, 28-day prescriptions require around 13 prescriptions [items] per year while 84-day prescriptions require around four).

### Geographical variation at CCG level across England

**Figure 1:**
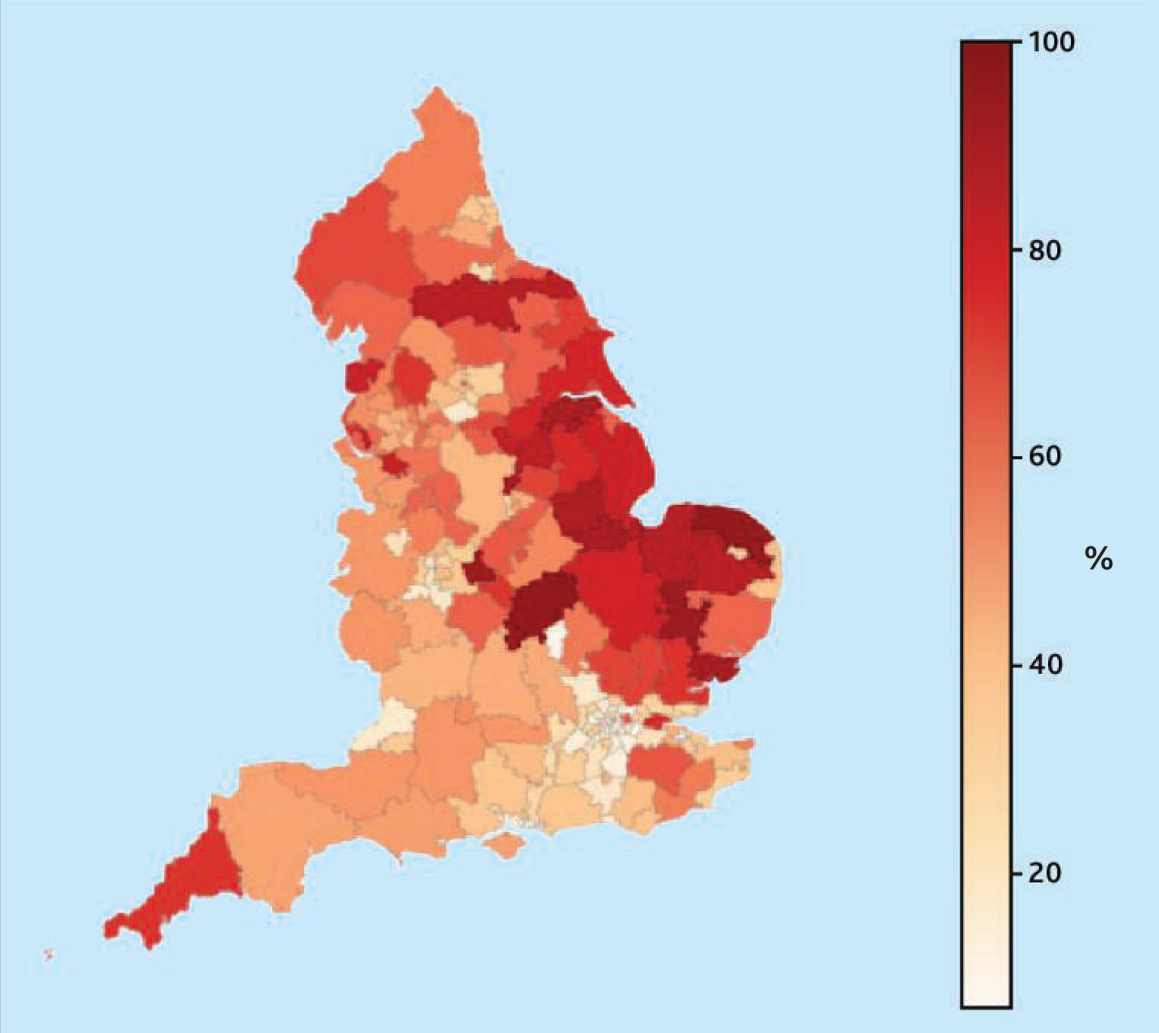
Twenty-eight-day prescribing proportion (%) for the basket of common medicines^a^ by each CCG in England.^b a^See Table 2. ^b^Equivalent maps for 56- and 84-day prescribing proportion (%) are shown in Supplementary Figure S1. CCG = clinical commissioning group.

The proportion of prescriptions issued for each duration (28, 56, and 84 days) was calculated out of the total issued for 28, 56, and 84 days, across CCGs in England. This geographical variation is displayed on choropleth maps (see Figure 1 and Supplementary Figure S1).

### Factors associated with prescribing durations

Two statistical models were used to examine practice factors associated with 28-day prescribing: a univariable Poisson model and a mixed-effects multivariable Poisson regression model. The dependent variable ‘28-day prescribing proportion’ was used, defined as the proportion of prescriptions that were issued for 28 days out of the total issued for 28, 56, and 84 days. Furthermore, publicly available data were used to specify the following variables for modelling:

- proportion of GP registered population aged ≥65 years;
- proportion of patients with a long-term condition;
- being a ‘dispensing practice’ with an in-house dispensing service (yes or no); and
- the practice EHR software system (EMIS, TPP SystmOne, Vision, or MicroTest).

These variables have previously been shown to be associated with variation in prescribing, therefore a univariable regression was created for each of them and all the variables were added into an exploratory mutually adjusted multivariable model. Random intercepts were specified in the model to accommodate variation between CCGs. Continuous variables were grouped *a priori* into quintiles for the analysis. Practices with missing data for a particular variable were not included in models containing that variable. From the resulting model, incidence rate ratios were calculated, with corresponding 95% confidence intervals (CIs). The level of missing data was determined and reported for each variable.

**Figure 2:**
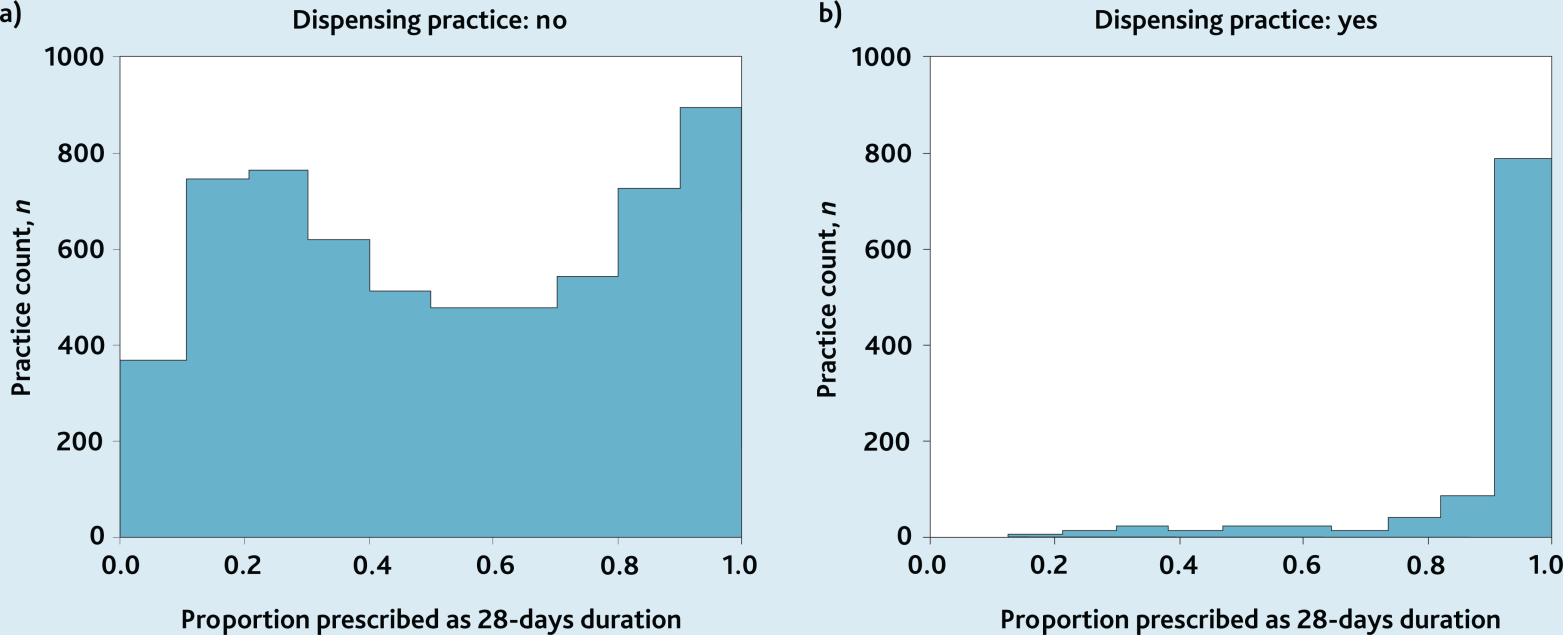
Histograms displaying the distribution of 28-day prescribing proportion for the basket of medicines across all practices in England, September 2018 to August 2019, split by dispensing status: a) non-dispensing; and b) dispensing.

A histogram of practice counts versus 28-day prescribing proportion (unadjusted figures) was plotted for factors that showed a significant association with 28-day prescribing (see Figure 2).

### Evaluation of EHR system user interfaces

One senior pharmacist tested the user interface of the two most widely used EHR systems in the study period (EMIS and SystmOne) by issuing prescriptions to a test patient and observing the prompts. The vendors of all four EHR systems were contacted to enquire about their default options for prescribing duration.

### Software and reproducibility

Data management was performed using Python 3 and Google BigQuery, with analysis carried out using Stata 13.2/Python 3. Data and all code for data management and analysis are archived online.^16^

### Patient and public involvement

The website OpenPrescribing.net is an openly accessible data explorer for all NHS England primary care prescribing data, which receives a large volume of user feedback from professionals, patients, and the public. This feedback is used to refine and prioritise informatics tools and research activities. Patients were not formally involved in developing this specific study design.

## Results

### Prescribing durations

Across the five medicines in the basket, 160 million prescriptions were issued in England during the 12-month study period (the 10 most commonly prescribed quantities are shown in Supplementary Figure S2). Of these, 133 million prescriptions were issued for 28-, 56-, or 84-day durations (see Table 3), totalling 5.11 billion tablets/capsules; 28-day prescriptions accounted for 48.5% of the total (2.5 billion tablets/capsules), 56-day prescriptions accounted for 43.6% (2.2 billion), while 84-day prescriptions accounted for only 8.0% (0.4 billion).

**Table 3:** Total number of items and quantity (tablets/capsules) issued as 28-, 56-, or 84-day durations across a basket of medicines^a^ typically prescribed once daily, in England, from December 2018 to November 2019

Quantity per item/durations, days	Total items, *n*	Total quantity, *n*	Proportion of total quantity^b^
**28**	88 410 515	2 475 494 420	48.5
**56**	39 714 191	2 223 994 696	43.6
**84**	4 834 340	406 084 560	8.0
**Total**	132 959 046	5 105 573 676	—

a See Table 2.

b Proportions do not sum to 100% due to rounding.

### Geographical variation across England

The 28-day prescribing proportion exhibited wide geographical variation, ranging from 7.2% to 95.0% across England’s 191 CCGs (median 45.6%, interquartile range [IQR] 28.1%–65.6%) (see Figure 1 and Supplementary Table S1).

### Factors associated with prescribing durations

Histograms show 28-day prescribing proportion versus dispensing status (Figure 2), EHR system supplier, and chronic condition quintiles (see Supplementary Figures S3 and S4).

The results of the regression analysis showed that each of the investigated factors were significantly associated with the 28-day prescribing proportion, with the exception of the percentage of patients aged ≥65 years (see Table 4). The strongest association was observed with dispensing status, where dispensing practices had a 64% higher 28-day prescribing proportion than non-dispensing practices (incidence rate ratio [IRR] 1.64, 95% confidence interval [CI] = 1.49 to 1.80). The percentage of patients with a chronic condition among the registered population had a dose-response relationship with the 28-day prescribing proportion, with the quintile of practices with the most patients with chronic conditions prescribing at a 27% higher rate than the lowest quintile (IRR 1.27, 95% CI = 1.12 to 1.44). There was also an association with software system, with TPP SystmOne having a higher 28-day prescribing proportion than EMIS (IRR 1.11, 95% CI = 1.02 to 1.21).

**Table 4:** Regression analysis. Twenty-eight-day prescribing proportion for the basket of medicines^a^ versus practice factors

Factor		Median proportion 28-day prescribing	Univariable poisson regression			Multivariable poisson regression		
			Rate ratio	95% CI		Rate ratio	95% CI	
**Proportion of patients aged ≥65 years, %**	0.0–10.8	44.36	Ref			Ref		
	10.9–15.4	55.38	1.10	0.99	1.23	0.97	0.86	1.09
	15.5–18.9	58.21	1.18	1.06	1.30	0.92	0.81	1.05
	19.0–22.7	62.89	1.23	1.11	1.37	0.90	0.79	1.02
	22.8–89.8	85.81	1.46	1.33	1.62	0.94	0.82	1.08
**Proportion of patients with a chronic condition, %**	10.0–43.8	36.55	Ref			Ref		
	43.9–49.4	51.31	1.19	1.07	1.33	1.09	0.97	1.23
	49.5–53.6	65.07	1.34	1.21	1.49	1.17	1.03	1.32
	53.7–58.3	70.22	1.42	1.28	1.58	1.20	1.06	1.36
	58.4–92.5	75.36	1.50	1.36	1.66	1.27	1.12	1.44
**EHR system**	EMIS	54.11	Ref			Ref		
	Microtest	93.51	1.40	0.97	2.03	1.05	0.65	1.69
	TPP SystmOne	70.27	1.15	1.08	1.23	1.11	1.02	1.21
	Vision	55.15	0.98	0.83	1.16	1.01	0.83	1.23
**Dispensing status**	Not dispensing	58.91	Ref			Ref		
	Dispensing	96.32	1.58	1.47	1.70	1.64	1.49	1.80

a See Table 2.

EHR = electronic health record.

Evaluating the user interfaces of the different EHR systems identified that default quantities were presented to prescribers when issuing prescriptions (although they can be easily ignored). Software vendors confirmed that defaults can be configured locally and that when EHR systems were initially implemented they followed the NHS drug tariff, which determines community pharmacy reimbursement.

## Discussion

### Summary

Across a small basket of common medications taken once daily, almost one-half (48.5%) of tablets prescribed over a 1-year period in England were in 28-day durations, 43.6% in 56-day (approximately 2 months) durations, and only 8.0% in 84-day (approximately 3 months) durations. There was very wide geographic variation (range 7.2%–95.0% 28-day prescribing proportion across CCGs). Dispensing status was the strongest predictor of shorter prescription duration; dispensing practices had a higher proportion of 28-day prescription durations than non-dispensing practices. The proportion of patients with chronic conditions and the EHR software used by a practice were also associated with prescription duration.

### Strengths and limitations

This data includes all prescribing in all typical general practices in England, thus minimising the potential for obtaining a biased sample. Real prescribing data were used, which are sourced from pharmacy claims, and therefore did not need to rely on surrogate measures. A small number of healthcare settings were excluded, such as walk-in centres, which typically do not issue repeat prescriptions for medicines. The data do not currently include dosage instructions on durations, so the study findings were limited to medicines typically issued as once-daily tablets/capsules. Our basket of five medicines centres around cardiovascular conditions and hypothyroidism treatments that are taken once daily, so findings may not be generalisable to other chronic conditions; for example, inhalers in asthma and chronic obstructive pulmonary disease.

The analyses included CCGs as during the study time period they were typically responsible for issuing local guidance to GPs on prescription duration; however, across the country there may be other organisations that have more influence in some local areas.

The study only includes data up to 2019. The manuscript development and submission were delayed by the outbreak of the COVID-19 pandemic and this limited the ability to conduct analysis on current data (both for prescribing and NHS organisations; that is, CCGs versus integrated care boards) and check the robustness of the multivariable modelling, through sequential adjustment and sensitivity analyses.

### Comparison with existing literature

To the authors’ knowledge, this article represents the first research using large-scale national data to estimate repeat prescription durations. Miani *et al* found that 3-month repeat prescriptions may be more cost effective and suggested that the NHS should encourage extension of repeat prescriptions from 28 days to 3 months.^9^ The present analysis indicates that 48.5% of prescriptions for commonly prescribed once-daily medications are for a 28-day duration, 43.6% for a 56-day duration, and 8.0% for an 84-day duration.

A practice's dispensing status had the strongest association with prescription duration, with dispensing practices almost exclusively prescribing 28-day courses for this basket of medicines. A potential explanation for this association is that dispensing practices are incentivised to prescribe medication with a 28-day duration due to the volume-based dispensing remuneration payment in place. The authors have previously demonstrated that dispensing practices were more likely to prescribe higher cost drugs, where they may negotiate lower prices while being reimbursed at a standard rate.^17^ Shorter durations may also be preferred by dispensing doctors for other reasons, including greater adherence to local guidance recommending 28-day prescribing durations, and for stock inventory management (for example, more storage space would be required to hold stock to supply longer durations).

The percentage of patients with chronic conditions was also associated with prescription duration. Practices that have more patients with a chronic condition will likely have more patients with multiple chronic conditions, likely requiring multiple treatments and more frequent monitoring and/or medication adjustments. Some medications have restrictions on their prescribed duration (for example, Schedule 2 controlled drugs are legally restricted to a 30-day supply) and the prescription duration for all co-prescribed medications is often determined by the one with the shortest acceptable duration (to avoid ordering synchronisation issues), so not all 28-day prescriptions will be suitable for extending to 56- or 84-day prescription durations. Additionally, for certain clinical conditions, medications, and individual patient circumstances, it will be necessary to have some flexibility in policy implementation.^18^

The study also identified that the EHR system used by a practice was associated with prescription duration, most notably TPP SystmOne had a higher 28-day prescribing proportion than EMIS. Both the EMIS and TPP SystmOne prescribing interfaces used the drug tariff recommended duration as the default (28 days for the basket of medicines in this study), but this could be locally configured if desired by a local primary care organisation in line with their repeat prescription policy. The authors’ previous work has shown that the user interface of each EHR system can substantially influence prescribing quality, safety, and cost.^19^–^22^ Individual GPs can choose which EHR software they use from national purchasing agreements; however, in practice, most GPs use the system recommended by their local NHS administrative area. As EHR system of choice is geographically clustered, it is possible that this effect may be driven by the adherence of GPs to the policies set by local primary care organisations and unrelated to the interface of the EHR system.

### Implications for research and practice

A recent report by the Chief Pharmaceutical Officer for NHS England to the Government on overprescribing identified that national organisations should support practices to improve consistency of repeat prescribing.^23^ This study clearly indicates inconsistency in repeat prescription duration across England, identifies opportunities for intervention at a policy level, and provides data to inform the policy debate on current practice.

Miani *et al* identified potential resource savings from increasing the default prescription duration.^9^ The present study makes no particular argument as to whether these recommendations are implemented but is presenting the data from national prescribing information to help inform policy making. The analysis identified that 49% of prescriptions are for a duration of 28 days and 44% are for 56 days. Accepting the underlying assumption of Miani *et al*,^9^ longer durations are likely to save time for clinicians and time for patients' interactions with the health service, in addition to broader benefits like reduced carbon emissions from trips to and from the pharmacy. However, the authors caution that substantial cash-releasing savings are unlikely to be reasonable within the current policy context. The same amount of medications need to be purchased by the NHS, and a small increase in wastage is possible from longer prescription durations. Dispensers (both community pharmacies and dispensing doctors) are paid on a per-item basis, and if there was a substantial shift to longer prescriptions it would likely lead to reduced income with minimal workload reductions. However, these data show substantial variation in current prescription duration patterns, suggesting the status quo may not be optimal or equitable to dispensers. In fact, current reimbursement structures may incentivise shorter prescription durations and that consequently reimbursement varies across the country, with limited regard to patient preference. The authors propose that these data can assist with repeat prescription policy development to provide a more consistent and equitable repeat prescribing experience for patients.

This study also lays the foundations for an economic evaluation based on real-world evidence using OpenPrescribing.net. There is further potential to link this to patient-level GP data on patient outcomes via secure platforms like OpenSAFELY, subject to appropriate permissions.

A limitation of this study is that it focused on a basket of once-daily medications. Developments in NHS data infrastructure implemented during the COVID-19 pandemic mean that dose syntax will be captured on primary care prescriptions in a machine-readable structured manner and this will soon be available for research analysis.^24^ This will allow the expansion of the basket of medicines to include a much larger range of medications including those with more complex dosing regimens. An analysis using patient-level data, for example in OpenSAFELY, could then provide information about health outcomes and NHS resource use associated with prescription duration for chronic conditions in the long term. Further, sensitivity analyses could explore the impact of including or excluding different medications, or sequentially adjusting by patient and practice factors to provide a more comprehensive view of the factors associated with variation in prescription duration. A targeted emulation trial may be a good alternative to expensive and long randomised control trials to harness the full potential of using such large datasets to estimate the potential consequences, both positive (for example, reduced dispensing fees, greater convenience, and saved time of patients and prescribers) and negative (for example, reduced benefits of medication review/monitoring and medication waste), of extending prescription duration from 28 days to either 56 or 84 days. This is in line with the call from Miani *et al* for better designed evaluation studies on this topic in the UK that use EHRs to facilitate data collection in near real time, which can be analysed by platforms such as OpenSAFELY.^9^ Such an emulated targeted trial could facilitate a pragmatic, unobtrusive, and low-cost evaluation across the whole country. Besides the traditional cost-per-quality-adjusted life year (QALY) estimates, such an evaluation should provide evidence about potential impact on health inequalities and patient experience, both of which are important when developing NHS policy. In addition, given the current NHS workload pressures, capacity issues of staff and primary care providers should also be considered. The multi-composite evidence of such an evaluation could support the implementation of new repeat prescription policies. The authors are now seeking collaborators and resources to undertake this analysis.

In conclusion, the present analysis of OpenPrescribing data showed that repeat prescriptions of 28-day durations are common for patients taking routine medications for chronic conditions, particularly in dispensing practices. This provides data to inform the policy debate on current practice. Configuration of EHR systems offer an opportunity to implement and evaluate new policies on repeat prescription duration in England.

## Supplementary Information



## Data Availability

The data source is publically available prescribing data. The code and data are available on Github: https://github.com/ebmdatalab/Rx-Quantity-for-Long-Term-Conditions
